# The analgesic effect of clonidine as an adjuvant in dorsal penile nerve block

**DOI:** 10.11604/pamj.2016.23.213.5767

**Published:** 2016-04-21

**Authors:** Jarraya Anouar, Smaoui Mohamed, Abidi Sofiene, Zouari Jawhar, Elleuch Sahar, Kolsi Kamel

**Affiliations:** 1 Anesthesiology Department, Hedichaker University Hospital, Sfax, Tunisia; 2 Hopital Kremlin Bicetre, Paris, France

**Keywords:** Dorsal penile nerve block, postoperative pain, circumcision

## Abstract

**Introduction:**

Dorsal penile nerve block (DPNB) is a commonly performed regional anesthetic technique for male circumcision. The aim of this study was to assess the analgesic effect of the adjunction of clonidine to bupivacaine 0.5% in this block.

**Methods:**

It was a prospective randomized double-blind clinical trial including 40 ASA1 boys aged from 1 to 4 years undergoing elective circumcision. Dorsal penile nerve block was performed under general Anesthesia. Patients were randomized in two groups: **Group 1 (G1):** received 0.1 ml/Kg of bupivacaine 0.5% with 1µg/kg of clonidine in each side. **Group 2 (G2):** received 0.1 ml/kg of bupivacaine 0.5% with placebo in each side. The failure of the DNPB was defined by the increase of heart rate by more than 25% comparing to baseline and in his case an intravenous injection of 20 µg/kg of alfentanyl was given. Post-operative pain was assessed by CHEOPS score.

**Results:**

A total of 40 patients were enrolled. Demographic parameters were similar in both groups. We noted no case of DNPB failure in this study. The supply for additional analgesia was seen in 12 patients in group 2 versus 3 cases in group 1. CHEOPS (Children's Hospital of Eastern Ontario Pain Scale) was significantly lower in group 1 from 2nd post operative hour until the 24th hour.

**Conclusion:**

Clonidine can be used in dorsal penile nerve block to improve and to prolong its analgesic effects after male circumcision.

## Introduction

Dorsal penile nerve block (DPNB) is a widely used regional anesthetic technique for male circumcision [1]. It is the regional anesthetic technique of choice for postoperative analgesia following male pediatric circumcision [1]. Despite the widespread use of sub pubic DPNB, a significant proportion of these patients require rescue opioid analgesia per operatively [2, 3]. As all circumcisions are performed in day care procedures and the administration of a strong opioid may delay patients discharge, enhancing the analgesic potency of the block seems to be very useful. The aim of our study was to improve the DPNB analgesic effect by the adjunction of clonidine as an adjuvant to bupivacaine 0.5% in this block.

## Methods

**Figure 1:**
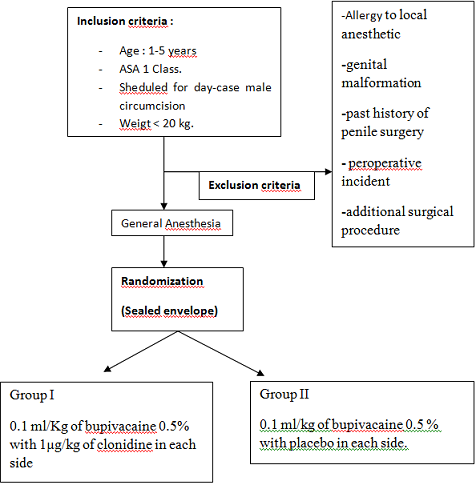
Consort diagram describing the inclusion and randomization process

The local ethics committee of Hedi Chaker university hospital approved this study, and parental consent was obtained for each case. Forty ASA I (American society of anesthesiologists) unpremedicated children, aged from 1 to 5 years (µg20 kg) and undergoing day-case male circumcision, were enrolled in this prospective, randomized, and double-blind study. Exclusion criteria were allergy to local anesthetic, genital malformation, past history of penile surgery, preoperative incident and additional surgical procedure other than circumcision. Each patient was randomly assigned to one of the two groups by drawing from a sealed envelope. Dorsal penile nerve block was performed in the operation room, with standard monitoring, under general anesthesia. General anesthesia was induced with Sevoflurane 6% and maintained with sevoflurane 3% in oxygen /air gas flow. The block was performed using the standard anatomical landmark technique. The penis was retracted gently in a caudate direction and the needle was inserted on either side of the midline just distal to the inferior ramus of the pubic bone. The needle was then advanced slowly, in a slightly medial and caudal direction, until a ‘pop’ was felt as it passed through Scarpa's fascia, and local anesthetic was deposited. We used pre-prepared syringes labeled “DPNB Study” Patients were randomized into 2 groups: **Group 1 (G1):** received 0.1 ml/Kg of bupivacaine 0.5% with 1µg/kg of clonidine in each side. **Group 2 (G2):** received 0.1 ml/kg of bupivacaine 0.5% with placebo in each side. The incision was performed at least 10 min after the block was completed. If, at the time of incision or during surgery, there was a rise in the heart rate or respiratory rate of >25% from baseline, an intravenous bolus of Alfentanyl (20 µg/kg) was given by an anesthetist, blinded to the injected solution in the block. As multimodal analgesia is the standard of care for postoperative pain in our department, supplemental analgesia of paracetamol IV perfusion (15 mg/kg) was provided systematically at the end of surgery. After surgery, patients were observed in PACU (Post Anesthesia Care Unit) for 6 hours by a nurse blinded to the study. In PACU, supplemental analgesia of intravenous nalbuphine increments of 0.2 µg/Kg was provided if the CHEOPS pain score was >7. Patients were discharged to home 6 hours after the admission in PACU if the Chung score was superior to 9. Pain score was assessed by parents and data were collected by phone after patients discharge. We should mention that on the day of the pre-anaesthetic visit, parents were taught to perform their role in the study and the use of CHEOPS score after discharge. At home, patients received oral paracetamol at the dose of 15mg/kg systematically and Ibuprophene was given if CHEOPS (Children's Hospital of Eastern Ontario Pain Scale) was superior to 7. Demographic data were compared using the Mann-Whitney U-test (age, body weight, size, anesthesia ans surgery duration). The numbers of patients requiring alfentanyl were compared using Yates continuity-corrected Chi-squared test. Pain scores and time to perform block were analyzed using Mann-Whitney U-test. A P value < 0.05 was taken as significant (Figure 1).

## Results

A total of 40 patients were enrolled in this study (20 patients in each group). Demographic parameters were similar in both groups (Table 1). We noted no case of DNPB failure in this study and no patient needed Alfentanyl per operatively. Per operative parameters as mean blood pressure and heart rate were comparable for both groups. After surgery, we noted no complications for this block and no patient needed Nalbuphine in PACU. CHEOPS score was inferior to 7 for all included patients during the first six post operative hours. CHEOPS (Children's Hospital of Eastern Ontario Pain Scale) was significantly lower in group 1 from H2 to H24 in comparison with group 2 (Table 2). The supply for additional analgesia after discharge was seen in 12 patients in group 2 versus 5 cases in group 1 (p < 0.05)

**Table 1:** Demographic data

	Group 1 (n = 20)	Group 2 (n = 20)	p value
Age (months)	30 ±3.12	25.2 ± 5	0.285
Weight (Kg)	14.5 ± 1.5	12.8 ± 2.1	0.111
Size (cm)	91 ± 5	87 ± 4	0.325
Surgery duration (min)	16 ± 2.4	17 ± 1.8	0.750
Anesthesia duration (min)	22 ±2.2	23 ±1.5	0.512

**Table 2:** Evolution of CHEOPS

CHEOPS	H0	H1	H2	H3	H4	H6	H12	H24
Group 1	5.8 (± 0.9)	5.2 (± 0.4)	4.5 (± 0.5)	4.5 (± 0.5)	4.6 (± 0.5)	4.5 (± 0.5)	5.0 (± 1.0)	5.8 (± 0.9)
Group 2	6.4 (± 0.8)	5.7 (± 0.9)	5.4 (± 0.5)	6.3 (± 0.4)	6.3 (± 0.5)	6.2 (± 1.1)	8.1 (± 0.7)	7.7 (± 0.4)
p value	0.083	0.054	<0.001	<0.001	<0.001	<0.001	<0.001	<0.001

H0: admission in post anesthesia care Unit; H1: 1 hour after admission in post anesthesia care Unit; H2: Two hours after admission in post anesthesia care Unit; H3: three hours after admission in post anesthesia care Unit; H4: four hours after admission in post anesthesia care Unit; H6: six hours after admission in post anesthesia care Unit; H12: 12 hours after admission in post anesthesia care Unit; H24: 24 hours after admission in post anesthesia care Unit; Cheops: Children's Hospital of Eastern Ontario Pain Scale

## Discussion

Male circumcision is a commonly practiced surgical procedure in our country (Tunisia) and DPNB is the preferred method of regional analgesia for circumcision, with excellent safety [4, 5]. Consequently, any intervention that would enhance the care of these patients would have a substantial impact. In our study we tried to improve and to prolong the analgesic effect of this technique by the adjunction of clonidine to the bupivacaine 0.5%. In our study we used the landmark technique and we noted no case of penile block failure. However, in recent years, the use of ultrasound has become popular in the performance of regional anesthesia, and several studies have shown the benefit of ultrasound over ‘blind'or ‘landmark’ techniques [6, 7]. However, others showed no difference between the two techniques for the DPNB [8]. Furthermore, our choice of local anesthetic was bupivicaine, compared with the choice of ropivicaine in other studies [9] reflecting differences in practice between institutions. Some studies [10, 11] recommended depositing a subcutaneous bleb of local anesthetic at the ventral base of penis as part of their technique of DPNB. Although this is not strictly part of the DPNB, previous studies [10, 11] have demonstrated that it is an essential part of providing complete local anesthesia to the penile tip. In our study, we performed the DPNB alone in both groups to avoid bias and we showed that it was sufficient to provide per and post operative analgesia. It is known that bupivacaine may be associated with adjuvant such as fentanyl or clonidine [12] to provide longer analgesia [13], but few trials studied its effect in DPNB for pediatric patients. A recent study [14] made in children hospital in Tunis including 36 patients undergoing penile surgery showed that the adjunction of clonidine to bupivacaine in DPNB improve post operative analgesia from H1 to H24 and reduce the need for opioids. These results are suitable with ours but it is not yet published.

## Conclusion

In this study, we showed the utility of the dorsal penile nerve bloc in male circumcision as it allowed us to avoid per operative opioid injections and as a consequence the early discharge of the patient. We also showed that the addition of clonidine to bupivacaine in the dorsal penile nerve block can provide better and longer postoperative analgesia. It can reduce the supply for additional analgesia.

### What is known about this topic

- Penile nerve block can reduce post circumcision pain.

### What this study adds

- Penile nerve block with clonidine improves and prolongs post circumcision analgesia.
